# Modified Gompertz equation for electrotherapy murine tumor growth kinetics: predictions and new hypotheses

**DOI:** 10.1186/1471-2407-10-589

**Published:** 2010-10-28

**Authors:** Luis E Bergues Cabrales, Juan J Godina Nava, Andrés Ramírez Aguilera, Javier A González Joa, Héctor M Camué Ciria, Maraelys Morales González, Miriam Fariñas Salas, Manuel Verdecia Jarque, Tamara Rubio González, Miguel A O'Farril Mateus, Soraida C Acosta Brooks, Fabiola Suárez Palencia, Lisset Ortiz Zamora, María C Céspedes Quevedo, Sarah Edward Seringe, Vladimir Crombet Cuitié, Idelisa Bergues Cabrales, Gustavo Sierra González

**Affiliations:** 1 Universidad de Oriente, Centro Nacional de Electromagnetismo Aplicado, Departamento de Bioelectromagnetismo, Grupo de Bioelectricidad, Av. Las Américas s/n. G.P. 4078. Santiago de Cuba 90400, Cuba; 2 Departamento de Física, Centro de Investigación y Estudios Avanzados del Instituto Politécnico Nacional, Laboratorio de Estimulación Magnética, Av. Instituto Politécnico Nacional 2508, Col. San Pedro Zacatenco, Ap. Post. 14-740, México, D.F. 07000, México 07360, Distrito Federal, México; 3 Universidad de Oriente, Centro de Biofísica Médica, Departamento de Biofísica. Santiago de Cuba 90500, Cuba; 4 Universidad de Oriente, Facultad de Ciencias Naturales, Departamento de Física. Calle Patricio Lumumba s/n. Santiago de Cuba 90500, Cuba; 5 Universidad de Oriente, Facultad de Ciencias Naturales, Departamento de Farmacia. Patricio Lumumba s/n. Santiago de Cuba 90500, Cuba; 6 Hospital Infantil Sur, Servicio de Oncohematología. Santiago de Cuba 90200, Cuba; 7 Dirección Municipal de Salud Pública. Servicio de Genética. Santiago de Cuba 90500. Cuba; 8 Hospital Oncológico Conrado Benítez, Servicio de Mastología. Santiago de Cuba 90500, Cuba; 9 Hospital Provincial Saturnino Lora, Servicio de medicina Interna. Santiago de Cuba 90500, Cuba; 10 Instituto Finley. Ave. 27 No. 19805, La Lisa, A.P. 16017 Cod. 11600. La Habana

## Abstract

**Background:**

Electrotherapy effectiveness at different doses has been demonstrated in preclinical and clinical studies; however, several aspects that occur in the tumor growth kinetics before and after treatment have not yet been revealed. Mathematical modeling is a useful instrument that can reveal some of these aspects. The aim of this paper is to describe the complete growth kinetics of unperturbed and perturbed tumors through use of the modified Gompertz equation in order to generate useful insight into the mechanisms that underpin this devastating disease.

**Methods:**

The complete tumor growth kinetics for control and treated groups are obtained by interpolation and extrapolation methods with different time steps, using experimental data of fibrosarcoma Sa-37. In the modified Gompertz equation, a delay time is introduced to describe the tumor's natural history before treatment. Different graphical strategies are used in order to reveal new information in the complete kinetics of this tumor type.

**Results:**

The first stage of complete tumor growth kinetics is highly non linear. The model, at this stage, shows different aspects that agree with those reported theoretically and experimentally. Tumor reversibility and the proportionality between regions before and after electrotherapy are demonstrated. In tumors that reach partial remission, two antagonistic post-treatment processes are induced, whereas in complete remission, two unknown antitumor mechanisms are induced.

**Conclusion:**

The modified Gompertz equation is likely to lead to insights within cancer research. Such insights hold promise for increasing our understanding of tumors as self-organizing systems and, the possible existence of phase transitions in tumor growth kinetics, which, in turn, may have significant impacts both on cancer research and on clinical practice.

## Background

Tumors are complex biological systems, and, in spite of great therapeutic advances, many of these still do not respond to treatment and lead to death. Part of the complexity of the problem is the sheer consequence of the tumor size and its histogenic characteristics. The cancer phenomenon continues to challenge oncologists. The pace of progress has often been slow, in part because of the time required to evaluate new therapies. To reduce the time to approval, new paradigms for assessing therapeutic efficacy are needed [1]. This requires the intellectual energy of scientists working in the field of mathematics and physics, collaborating closely with biologists and clinicians. This essentially means that the heuristic experimental approach, which is the traditional investigative method in the biological sciences, should be complemented by a mathematical modeling approach [2].

Significant research has been done in the modeling of tumors using theoretical models and computer simulations in order to describe and predict various aspects of tumor growth kinetics (TGK). Predicting tumor growth is important in the planning and evaluation of screening programs, clinical trials, and epidemiological studies, as well as in the adequate selection of dose-response relationships regarding the proliferative potential of tumors [2-5].

The biological behavior of a malignant tumor is highly influenced by its growth rate, which is determined by many intratumoral and micro-environmental factors. The space-time permanent growth is probably the most characteristic feature of a malignant tumor.

Further advancement in mathematical modeling of TGK critically depends on a thorough testing of proposed models against new data as they become available with the development of experimental techniques [3-6]. Thus, it is apparent that theoretical mathematical models are needed to study cancer.

In electrotherapy (ET) with low-level direct electrical current (DEC), mathematical modeling has not been used. ET was revolutionary when first introduced and is a promising surgical technique for destroying tumors. It has been shown to be a very useful, alternative tool against cancer. Preclinical and clinical studies have shown that ET is simple, safe, effective, and, minimally traumatic, with few side effects. It provides a method for treating solid cancers that are conventionally inoperable, those that cannot be resected after thoracotomy, and those that are not responsive to chemotherapy or radiotherapy [7-10]. Similar results have been reported by our research group [11-15].

Although preclinical and clinical studies have shown that ET has a marked antitumor effect, it is not widely used in clinical practice. The reason is that ET is not a well-established therapy due to the lack of a standardized method and unclear knowledge concerning the mechanisms involved. As a result an optimal electrode distribution has not been determined for ET, nor has the dose-response relationship been established. For this reason, we pay special attention to these two factors [16,17].

Camué *et al*. [14] experimentally report that an increase in DEC intensity increases its antitumor effectiveness, and that Ehrlich and fibrosarcoma Sa-37 tumors have a DEC threshold for which their complete remission is reached. These results have been theoretically corroborated through the use of a modified Gompertz equation (MGE), which has a good prediction capability to describe both unperturbed and DEC-perturbed TGK [17].

Many intrinsic processes that occur in both unperturbed and DEC-perturbed TGK are unknown. We believe that the MGE can be used as a tool to reveal some of these processes in order to improve DEC effectiveness. The aim of this paper is to describe the complete growth kinetics of unperturbed and DEC-perturbed fibrosarcoma Sa-37 tumors through the MGE in order to generate useful insights into the mechanisms that underpin this devastating disease. In this study, we analyze this model taking into account the experimental data reported in [14] for fibrosarcoma Sa-37 tumor. Also, we discuss the current limitations and potential implications of this model for further TGK research. It is important to note that the results reported in [14] and [17] support this paper.

## Methods

This study is approved by the Committees of Ethics of the National Center of Electromagnetism Applied (CNEA) and the Conrado Benitez Oncologic hospital, Santiago de Cuba, Cuba.

### Complete growth kinetics for unperturbed and DEC-perturbed tumors

Our experiences in preclinical and clinical studies have indicated that DEC-treated TGK is complex, with two well-defined regions (REG-I and REG-II). REG-I (defined before DEC treatment is performed) includes the initial time of tumor cell inoculation (t = 0 days) up to the moment that tumor is perturbed by a DEC stimulus, which occurs when it reaches a volume V_o _(initial volume selected by the therapist). REG-II (defined after DEC treatment is performed) includes the time at which the tumor is perturbed by DEC stimulus up to the end of the experiment.

In preclinical studies, the end of the experiment is fixed by the researcher, whereas in clinical studies, it can occur at multiple events: 1) the patient dies, 2) the patient leaves the clinical trial, or 3) the patient is completely cured [9,10,15]. It is important to point out that this REG-II is only reported in the field of ET in cancer [9-15,18-21].

### Electrochemical treatment

Once fibrosarcoma Sa-37 tumors have reached approximately V_o _= 0.5 cm^3 ^in BALB/c mice, four platinum electrodes are inserted into their bases and a single-shot electrotherapy is supplied. V_o _is reached 15 days after viable tumor cells are inoculated in the dorsolateral region of the animals. Four groups (one control group and three treated groups), each consisting of ten mice, were randomly formed: the control group (CG), a treated group with 36 C/cm^3 ^(18 C in 0.5 cm^3^) and 6.7 mA for 45 min (TG1), a treated group with 63 C/cm^3 ^(31.5 C in 0.5 cm^3^) and 11.7 mA for 45 min (TG2), and a treated group with 80 C/cm^3 ^(40.0 C in 0.5 cm^3^) and 14.8 mA for 45 min (TG3). The experimental details are discussed by Camué *et al*. [14].

### Modified Gompertz equation

A feature of the MGE is that it is developed after the experiments. It is implemented in order to fit the experimental data corresponding to REG-II for the Ehrlich and fibrosarcoma Sa-37 TGK [17], given by

(1) $$V^{*}(t')=V_{o}\;e^{\left(\frac{\alpha^{*}}{\beta }\right)\left(1-e^{-\beta \;t}{}^{'}\right)}$$

where

(2) $$\alpha^{*}=\left[a_{1}\;\left(1-e^{-\gamma \;t}{}^{'}\right)+a_{2}\right]\;\alpha ,$$

with

(3) $$a_{1}=\left(\frac{i}{i_{o}}\right)\left(2-\frac{i}{i_{o}}\right),$$

and

(4) $$a_{2}=(1-\frac{i}{i_{o}}),$$

All parameters involved are real and positive. V*(t') represents the tumor volume (TV) at time t' after DEC treatment. The parameter α (α > 0) is the intrinsic growth rate of the tumor related to the initial mitosis rate. The parameter β (β > 0) is the growth deceleration factor related to the anti-angiogenic process. The parameter α* is the modified tumor growth rate due to DEC action. *i *(*i *> 0) is the DEC intensity that flows through the tumor by the application of an external electric field. *i*_*o *_(*i*_*o *_> 0) is the polarization current (or electric current distributed into the tumor by DEC action). The parameter γ is the first-order exponential decay rate of the net effect induced in the solid tumor after the DEC is removed and its inverse is the decay constant (or decay time) that characterizes the duration of such an effect. *a*_*1 *_and *a*_*2 *_are dimensionless parameters that depend only on the (*i*/*i*_*o*_) ratio.

The results obtained from fitting the experimental data for REG-II of fibrosarcoma Sa-37 TGK for CG, TG1, TG2, and TG3 are shown in Table 1. The numerical information is obtained upon fitting the individual tumor growth data and then fitting the data for each experimental group. The mean values and standard errors of these optimized parameters after fitting were computed [17].

**Table 1:** Mean ± standard error of the parameters obtained from fitting the experimental data of the growth curve of fibrosarcoma Sa-37 tumors using the MGE

Groups*	**α (days**^**-1**^**)**	**β (days**^**-1**^**)**	**γ (days**^**-1**^**)**	*i_o _*(mA)
CG	0.513 ± 0.009	0.262 ± 0.006	0.000 ± 0.000	0.000 ± 0.000
TG1	1.793 ± 0.028	0.142 ± 0.006	0.184 ± 0.003	4.342 ± 0.007
TG2	1.584 ± 0.030	0.076 ± 0.002	0.107 ± 0.001	4.342 ± 0.007
TG3	0.006 ± 0.001	0.207 ± 0.002	0.189 ± 0.016	1.080 ± 0.210

*CG: control group. TG1: treated group with electrical current of 6.7 mA. TG2: treated group with 11.7 mA. TG3: treated group with 14.8 mA. Each experimental group consisted of 10 mice [14,17], which are exposed for 45 minutes.

### Interpolation of data corresponding to REG-II of fibrosarcoma Sa-37 TGK

Interpolation of experimental data corresponding to REG-II of fibrosarcoma Sa-37 TGK was developed. From an experimental point of view, to perform such a study, the information in ET is reported in terms of a non equidistant time dependence of TV (V*(t') vs. t' plot, named TV plot) spaced by one day or more. As a result, the TGK details are not revealed. At the experimental level, it is difficult to show the TGK for a small time step like one day because such a study would be cumbersome, expensive in resources, time-consuming, and requiring excessive handling of animals, which is not permitted by the ethics code care and use of Laboratory Animals Committee. For this reason, we interpolated the experimental data corresponding to REG-II for this tumor type using different time steps, Δt (1, 1/3, 1/8, 1/24, and 1/48 days). In this case, we take into account the mean values of each parameter in the MGE for each experimental group (Table 1).

### Reconstruction of REG-I for fibrosarcoma Sa-37 TGK

In the ET framework, neither experimental nor theoretical reports have taken into account REG-I of TGK which, for the former, can be very important for understanding the fibrosarcoma Sa-37 natural history before DEC treatment and its future influence on therapeutic effectiveness after DEC treatment. For this reason, we reconstructed this first region using an extrapolation method (to find unknown values for TV in points that are outside the typical studied range) for each Δt. In order to obtain the complete TGK for CG, we substitute t' with (t-τ) in Equation 1, keeping in mind the α, β, and V_o _parameters (Table 1) and the interpolated experimental data for REG-II. In this case, τ is a time delay that represents the time interval from the point at which the tumor cells are inoculated in the host until the solid tumor reaches V_o_.

The considerations included in the MGE are:

1. For unperturbed tumors (α* = α for *i *= 0), α in Equation 1, is constant during TGK. In this case, the MGE coincides with the conventional Gompertz equation [1,17,22,23].

2. REG-I for fibrosarcoma Sa-37 TGK is the same for CG, TG1, TG2, and TG3. As a result, *α *in Equation 1, is the same for all of the experimental groups. This assumption has been experimentally corroborated, since the tumors in CG, TG1, TG2, and TG3 reach V_o _at approximately the same time τ (τ = 15 days) [14].

The MGE can be rewritten in the form

(5) $$V^{*}(t)=\left\{ \begin{array}{l} V_{o}\;e^{\left(\frac{\alpha }{\beta }\right)\left(1-e^{-\beta (\;t-\tau )}\right)}\;0\leq t\leq \tau \\ V_{o}\;e^{\left(\frac{\alpha^{*}}{\beta }\right)\left(1-e^{-\beta \;(t-\tau )}\right)\;\tau \leq t\leq \tau +t'} \end{array}\right.$$

where t is the time that elapses from the initial moment at which tumor cells are inoculated in the host (t = 0 days) up to the end of the experiment. t' is the time that elapses from the moment of DEC application up to the end of the experiment.

### Graphical strategies for the analysis of TGK of the experimental groups

Different graphical strategies are used in order to obtain further time-dependent information for both untreated and DEC-treated TGK that is not revealed in a simple TV plot. For this reason, we use the following plots: first derivative of tumor volume (FDTV) versus t, named the FDTV plot (or dV*(t)/dt vs. t plot); TV dependence of FDTV, named FDTV-TV plot (or dV*(t)/dt vs. V*(t) plot), the time consecutive dependence on TV plot, named CTV plot (or V*(t) vs. V*(t-Δt) plot); and the modules and log-log plots for TV and FDTV-TV in order to analyze whether REG-I and REG-II for TG3 are the same.

It is important to point out that the results shown in this paper are in long format (scaled fixed point with 15 digits after the decimal point).

## Results

### Analysis of complete unperturbed fibrosarcoma Sa-37 TGK

**Figure 1:**
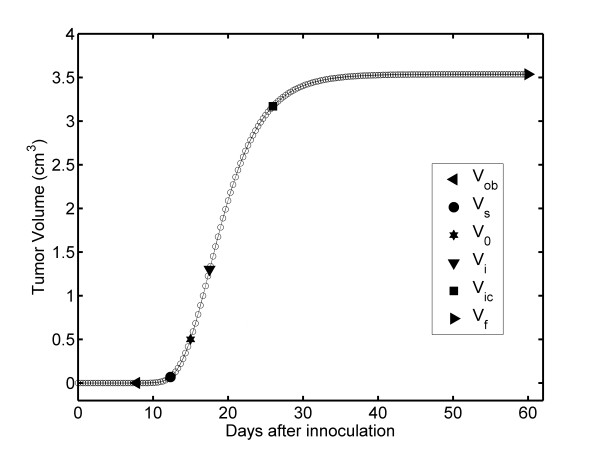
**Time dependence of TV: Unperturbed fibrosarcoma Sa-37 TGK (CG) for the parameters *i *= 0 mA, α = 0.513 days^-1^, β = 0.262 days^-1^, V_o _= 0.5 cm^3 ^and a time step of Δt = 1/3 days**.

The complete growth kinetics of unperturbed fibrosarcoma Sa-37 tumors are generated by interpolation of the experimental data for REG-II and the extrapolation process for REG-I using Equation 5 with values for α, β, τ, and V_o _from the CG (Table 1). TGK exhibits a characteristic S shape with three stages (SI, SII, and SIII), which are well defined for all Δt values, as shown in Figure 1 for Δt = 1/3 days.

The results show that SI is nonlinear and that there are two intersection points that separate each of the stages. The first point (V_s _in Figure 1) is obtained from the interception between SI and SII, and it represents the beginning of SII (TGK is triggered). The second point (V_ic _in Figure 1) is obtained by the interception of SII and SIII, representing the beginning of SIII (TV tends to a limit value, V_f_). V_ic _represents the irreversible TV from which it growth up to V_f_. The V_s_, V_ic_, and V_f _points are characterized by an ordered pair (t, TV) and are estimated as (12.34 days, 0.069 cm^3^), (25.99 days, 3.169 cm^3^) and (60 days, 3.536 cm^3^) for all values of Δt, respectively.

The interpolation and extrapolation processes reveal that unperturbed fibrosarcoma Sa-37 TGK has a point of inflection, V_i _at (17.56 days, 1.301 cm^3^). This value may also be analytically corroborated by making *i *= 0 and setting the second derivative of Equation 1 to zero. V_i _is a point in the TGK at which the curvature changes from concave upwards (positive curvature) to concave downwards (negative curvature). Additionally, these processes predict three other TV values, which are observed in the experiment: V_o _= 0.5 cm^3 ^at 15 days; 0.02 cm^3 ^at 11.29 days; and 0.03 cm^3 ^at 11.60 days [14]. In preclinical studies, our experience shows that 0.02 cm^3 ^is the smallest measurable TV, designated as V_m _[11-14]. V_m _for fibrosarcoma Sa-37 tumors is experimentally observed at 12 days [14]. The difference in time is 0.71 days, which is not significant from an experimental point of view.

We macroscopically observe the first non-zero volume, V_ob_, for fibrosarcoma Sa-37 at 8 days (Figure 1). This tumor size is observable and palpable, but not measurable. Equation 5 estimates V_ob _to be 0.000016 cm^3 ^(0.031 cm in diameter) for this time value.

**Figure 2:**
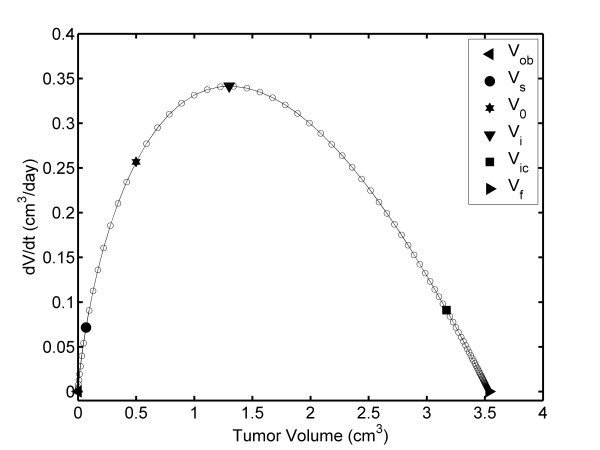
**TV dependence of the FDTV: Unperturbed fibrosarcoma Sa-37 TGK (CG) for the parameters *i *= 0 mA, α = 0.513 days^-1^, β = 0.262 days^-1^, V_o _= 0.5 cm^3 ^and a time step of Δt = 1/3 days**.

The FDTV-TV plot (Figure 2) shows that TV increases between V_obs _and V_f_; however, FDTV increases from V_s _to V_i _and then decreases to zero (from V_i _to V_f_). Figure 2 illustrates that reaches its maximum value, FDTV_max _when TV reaches V_i_. The ordered pair (V_i_, FDTV_max_) is (1.301 cm^3^, 0.341 cm^3^/day), which is observed at 17.56 days.

### Analysis of REG-II DEC-perturbed TGK for TG2

**Figure 3:**
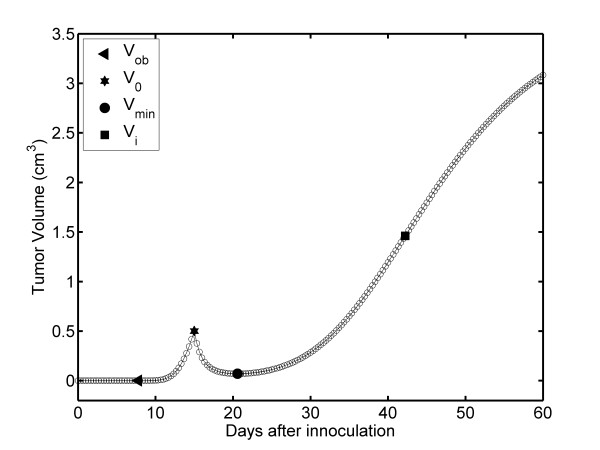
**Time dependence of TV: DEC-perturbed fibrosarcoma Sa-37 TGK (TG2) for the parameters *i *= 11.7 mA, α = 1.584 days^-1^, β = 0.076 days^-1^, γ = 0.107 days^-1^, *i*_*o *_= 7.431 mA, and V_o _= 0.5 cm^3 ^and a time step of Δt = 1/3 days**.

We directly present the results for REG-II TGK for tumors treated with DEC for 45 minutes because the REG-I is similar to that of the CG. The complete fibrosarcoma Sa-37 TGK treated with 11.7 mA (TG2) is shown in Figure 3, for Δt = 1/3 days, in agreement with other values of Δt = 1, 1/8, 1/24, and 1/48 days (results not shown). This figure reveals that REG-II TGK (from V_o _up to end of the experiment) is characterized by two sub-regions (REG-IIa and REG-IIb). In REG-IIa, TV decreases from V_o _to its minimum volume, V_min_, whereas in REG-IIb, it increases from V_min _until the end of the experiment. Additionally, V_min _is estimated by the interception of REG-IIa and REG-IIb, resulting in a value of 0.0698 cm^3 ^that is reached at 20.58 days, for all Δt values, as shown in Figure 3, for Δt = 1/3 days. V_min _is analytically corroborated through the following transcendent equation, constructed by minimizing V*(t') in Equation 1, given by

(6) $$a_{1}\;\gamma \;e^{-\gamma \;t}\;+\;\beta \left(a_{1}\;+\;a_{2}\right)\;\;e^{-\beta \;t}\;-a_{1}\;\left(\gamma \;+\;\beta \right)\;e^{-\gamma \;t}\;e^{-\beta \;t}\;\;=0$$

For this, we substitute the values of α, β, γ, *i*, and *i*_*o *_for TG2 (Table 1) in Equation 6. V_min _is experimentally observed to be 0.07 cm^3^, reached at 21 days after the inoculation process. The differences between the estimated and analytical values are 0.0002 cm^3 ^for TV and 0.42 days for time, neither of which are significant at the experimental level.

**Figure 4:**
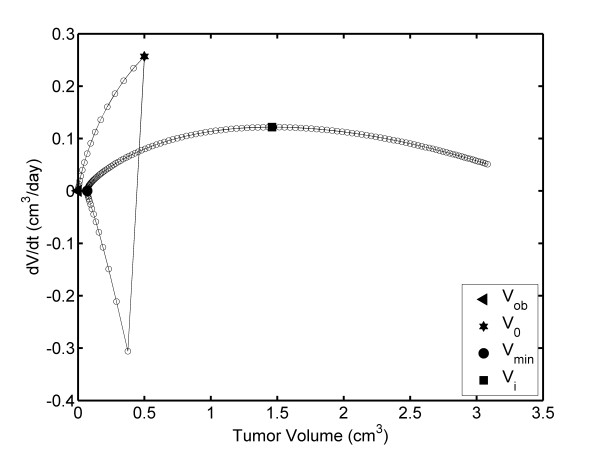
**TV dependence of the FDTV: DEC-perturbed fibrosarcoma Sa-37 TGK (TG2) for the parameters *i *= 11.7 mA, α = 1.584 days^-1^, β = 0.076 days^-1^, γ = 0.107 days^-1^, *i*_*o *_= 7.431 mA, and V_o _= 0.5 cm^3 ^and a time step of Δt = 1/3 days**.

Figure 4 shows that when TV decreases from V_o _to V_min_, FDTV first decreases from 0.5 to 0.376 cm^3 ^(positive slope) and then it increases from 0.376 to 0.069 cm^3 ^(negative slope). The FDTV values for 0.376 and 0.069 cm^3 ^are - 0.3069 cm^3^/days (at 15.33 days) and 0.000068 cm^3^/days (at 20.58 days), respectively. The values of - 0.3069 cm^3^/days corresponds to the minimum negative value of FDTV, FDTV_min_, which is observed at 15.33 days and between V_o _and V_min _(Figure 4); however, this is not revealed in the TV plot (Figure 3). The TV correspondent to FDTV_min _in Figure 4, reveals that when the TV reaches V_min_, it always increases from V_min _up to V_f_; however, FDTV increases from V_min _up to V_i _(predicted as (1.461 cm^3^, 0.121 cm^3^/days) and reached at 42.22 days). Then, FDTV decreases to zero from V_i _up to V_f_.

### Analysis of REG-II DEC-perturbed TGK for TG3

**Figure 5:**
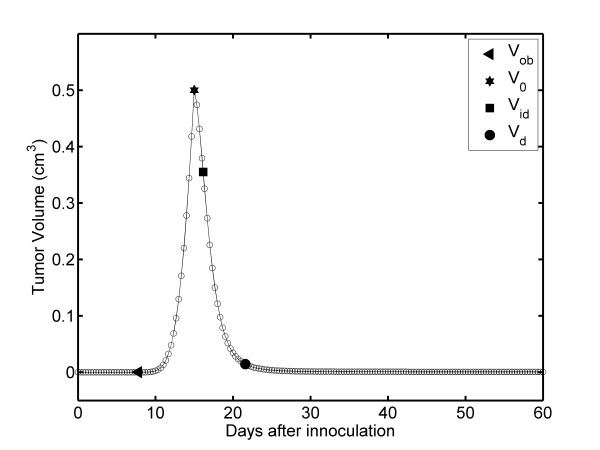
**Time dependence of TV: DEC-perturbed fibrosarcoma Sa-37 TGK (TG3) for the parameters *i *= 14.8 mA, α = 0.006 days^-1^, β = 0.207 days^-1^, γ = 0.189 days^-1^, *i*_*o *_= 1.080 mA, and V_o _= 0.5 cm^3 ^and a time step of Δt = 1/3 days**.

A completely different picture is observed in REG-II of the DEC-perturbed fibrosarcoma Sa-37 TGK for TG3, as shown in Figure 5. This TGK is characterized by a decrease of TV from V_o _to 0 cm^3^, with two well-defined sub-regions (REG-IIc and REG-IId). In REG-IIc, TV rapidly changes from V_o _to TV from which begins its complete destruction, named V_d _(estimated as 0.014 cm^3 ^at 21.58 days); however, in REG-IId, it slowly varies from V_d _to zero, reached at 30 days after the inoculation process (15 days after DEC treatment), as shown in Figure 5, for Δt = 1/3 days. These results are in agreement with those obtained for the other values of Δt. This figure reveals that in REG-IIc, there is a point of inflection V_id_, which is predicted at (16.15 days, 0.355 cm^3^).

**Figure 6:**
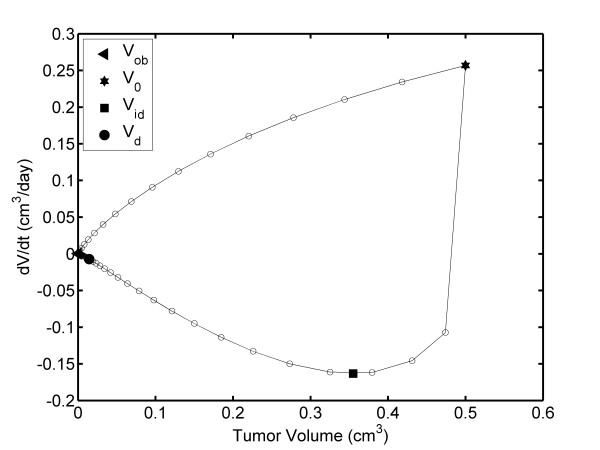
**TV dependence of the FDTV: DEC-perturbed fibrosarcoma Sa-37 TGK (TG3) for the parameters *i *= 14.8 mA, α = 0.006 days^-1^, β = 0.207 days^-1^, γ = 0.189 days^-1^, *i*_*o *_= 1.080 mA, and V_o _= 0.5 cm^3 ^and a time step of Δt = 1/3 days**.

Figure 6 reveals, that TV and FDTV decrease from V_o _to V_id _(estimated as 0.355 cm^3^, -0.163 cm^3^/day) at 16.15 days (positive slope), and then both magnitudes decrease up to a state of the tumor characterized by the ordered pair (0.014 cm^3^, - 0.0072 cm^3^/day) at 21.58 days (negative slope). TV and FDTV abruptly decrease to zero for TV smaller than V_d _(negative slope).

**Figure 7:**
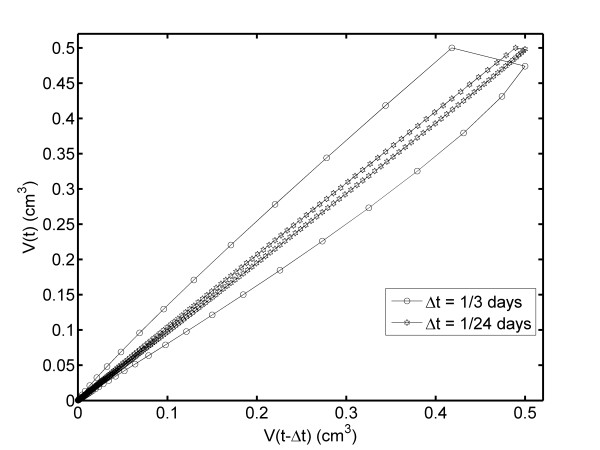
Time consecutive dependence of the TV plot (V*(t) vs. V*(t-Δt) plot) for Δt = 1/3 and 1/24 days.

**Figure 8:**
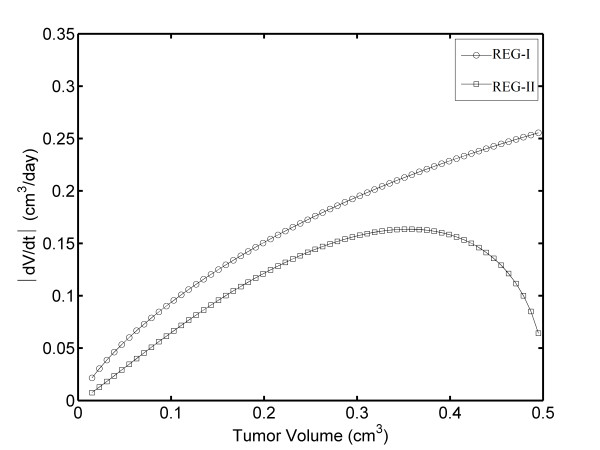
**Analysis conducted by separating REG-I and REG-II in a plot of module of FDTV versus TV**.

We propose a CTV plot in order to demonstrate whether the tumor can be completely reversible, as shown in Figure 7. In this figure, a closed loop appears for Δt = 1/3 and 1/24 days, being narrower for Δt = 1/24 days. This figure reveals that REG-I and REG-II for fibrosarcoma Sa-37 TGK are not symmetric. In addition, an analysis of the FDTV module versus TV plot (Figure 8) and the log-log plot (log TV versus log t, log FDTV versus log t, and log V(t) versus V(t-Δt)) are conducted to demonstrate whether REG-I and REG-II are proportional. The figures indicate that these two regions are not symmetrical or equal.

Eliminating the nonlinear part in both REG-I and REG-II shown in Figure 8, we may fit each one of these regions to a straight line. For REG-I, the slope ± its error and the intercept ± its error are 0.724 ± 0.011 and - 0.729 ± 0.024, respectively. These respective parameters are 0.992 ± 0.031 and - 0.524 ± 0.068, for REG-II. The ratio between the slopes is 1.37.

In the CG, the CTV plot shows that V(t) increases with increasing V(t-Δt), as expected. Additionally, the CTV plot for TG2 reveals that both V(t) and V(t-Δt) decrease to 0.376 cm^3 ^beyond V_o_. Then, V(t) increases with decreasing V(t-Δt) until reaching V_min_. Beyond this value, both V(t) and V(t-Δt) increases.

The patterns shown in Figure 3 and Figure 4 for TG2 are similar to those for TG1. Furthermore, the results obtained for fibrosarcoma Sa-37 TGK are similar to those obtained for Ehrlich tumors in the three experimental groups. For this reason, in the present study, such results are not included.

## Discussion

We show that the macroscopic behavior of both untreated and DEC-treated fibrosarcoma Sa-37 TGK can be realistically modeled using Equation 5. For this, we use previous experimental data for CG, TG1, TG2, and TG3 [14]; the parameters are obtained from fitting these data (Table 1) [17], and both interpolation and extrapolation methods for different time steps Δt (1; 1/3; 1/8; 1/24; and 1/48 days) are used.

### Unperturbed fibrosarcoma Sa-37 TGK

The TV plot corroborates that the complete untreated fibrosarcoma Sa-37 TGK (*i *= 0) exhibits an S shape with three well-defined stages (SI, SII, and SIII) (Figure 1). SI is common to each experimental group, and it is associated with the establishment of the tumor in the host. SII is related to rapid tumor growth. SIII of this kinetic shows slow tumor growth and its behavior towards V_f _(asymptotic value).

In SI, V_ob _for fibrosarcoma Sa-37 tumor at 8 days is experimentally observable and palpable but not measurable [14]; however, Equation 5 predicts this value. Nerterets *et al*. [24], reported tumor diameters below 0.025 cm via imaging with X-ray phase-contrast micro-CT in-line holography. The extrapolation of SI estimates a tumor size of 0.0000082 cm^3 ^to be reached at 7.79 days, with the first approximation assumption that 0.025 cm^3 ^is the smallest volume measured for all tumor types. The differences between these values and those estimated for this tumor type are 0.0000078 cm^3 ^for TV and 0.21 days for time, which are not significant at the experimental level.

Experimentally, TV is measured with a vernier caliper with a precision of 0.005 cm, and the thickness of the mouse skin (between 0.1 and 0.2 cm) is taken into account. Our experience indicates that above 0.02 cm^3^, the mouse skin thickness is negligible as compared with the tumor size [11-14]. Below 0.02 cm^3^, this thickness is comparable and larger than the tumor size, being more evident when the TV approaches V_ob_.

Equation 5 is continuous and smooth for all t (from t = 0 up to the end of the experiment), in contrast with the experiment. The tumor sizes are smaller than 10^-6 ^cm^3^, below V_ob_, which cannot be observed or measured with any of the current experimental techniques for measuring TV, and therefore, to a first approximation, the sizes are considered as zero, in agreement with our experimental observations [11-14]. This suggests that REG-I consists of two parts: from t = 0 up to t_ob _(t_ob_: observable time, in days, for which V_ob _is observed) and from t_ob _up to τ. As a result, Equation 5 can be rewritten as

(7) $$V^{*}(t)=\{\begin{array}{ll} 0 & for\;0\leq t\leq t_{ob}\\ V_{o}\;e^{\left(\frac{\alpha }{\beta }\right)\left(1-e^{-\beta (\;t-\tau )}\right)} & for\;t_{ob}\leq t\leq \tau \\ V_{o}\;e^{\left(\frac{\alpha^{*}}{\beta }\right)\left(1-e^{-\beta \;(t-\tau )}\right)} & {}^{for\;\tau \leq t\leq \tau +t'} \end{array}$$

Equation 7 suggests that the MGE is continuous for t ≥ t_ob_. Our experience indicates that V_ob _and t_ob _depend on the tumor histogenic characteristics, the host type, and the initial concentration of tumor cells inoculated in the host [11-14].

We experimentally observe that fibrosarcoma Sa-37 solid tumors are spheroids between 8 and 10 days (SI of TGK), which are also palpable and observable but not measurable. Our model predicts that TV at 10 days is 0.0025 cm^3 ^(0.17 cm in diameter). It is surprising that this volume range (0.031 to 0.17 cm in diameter) for which the tumor is spherical coincides with that reported by other authors for the avascular phase (0.025 up to 0.2 cm in diameter) [5,25-32]. Our model estimates that a tumor 0.2 cm in diameter (0.0042 cm^3^) is reached at 10.28 days. The differences for volume and time are 0.0017 cm^3 ^and 0.28 days, which are not significant at the experimental level.

The fact that the tumors are spheroids (between 0.000016 and 0.0025 cm^3^) may be explained by a central force field of the Coulomb type due to the fact that the cancer cells are negative charged [33]. It is important keep in mind that a force field is central if and only if it is spherically symmetric. An increase in the tumor cell number occurs when the tumor grows, and as a result, these cells are closer. Since they have the same electrical charge, they are repelled and the tumor is deformed, a fact that explains why the tumor has an ellipsoidal shape after 10 days.

The results show that TGK for SII changes quickly at first (from V_s _up to V_i_: concave upwards) and then slowly (from V_i _up to V_ic_: concave downwards). This pattern occurs because FDTV first increases and then decreases with increasing TV. In the first case (when both TV and FDTV increase), several factors are involved, such as local growth that is facilitated by enzymes (e.g., proteases) that destroy adjacent tissues and, tumor angiogenesis factors that are produced to promote formation of the vascular supply required for further tumor growth, among others [1]. In the second case (when FDTV decreases with increasing TV), the tumor itself generates different mechanisms that oppose its own growth (i.e., anti-angiogenic substances). If the tumor does not generate such mechanisms, its growth would be exponential and, as a consequence, the tumor-host relationship would be broken, which is not observed in oncological practice [1]. This may indicate that unperturbed tumors intelligently regulate their own growth. This means that the tumor self-organizes, and as a result, new emergent variables appear in order for the tumor to grow, evade the immune system, and achieve maximum survival.

The FDTV behavior may suggest that the tumor doubling time and α are not constant during unperturbed fibrosarcoma Sa-37 TGK, in agreement with Steel [22]. This result is in contrast with the fact that these two kinetic parameters are constant during all TGK, as we assume in this paper and as reported previously by our group [17] and other authors [1,23]. Additionally, the TV dependence of FDTV indicates that, V_i _may have important implications in DEC planning, if we take into account the fact that the tumor is more sensitive to DEC than healthy tissue [7-15,18-21], the Steel equation [22], and the results of Smith *et al*. [34].

In SIII, the tumor behavior is explained by the fairly slow rate of growth due to the amount of nutrients and O_2 _needed for quick expansion of the tumor [1,22].

Both interpolation and extrapolation methods estimate V_m_, V_s_, V_o_, and V_f _with good accuracy as well as their respective times, which are experimentally observed [14]. This is reasonable because the differences between the experimental and theoretically predicted values for these volumes and times at the experimental level are not significant. Furthermore, these methods predict V_i _and V_ic _and their respective times, which are not available from a TV plot. These points may have important implications in TGK and tumor treatment. The existence of V_ic _establishes the irreversibility of TGK.

Our experience in preclinical studies indicates that a good DEC effectiveness is obtained for TV smaller than 1.5 cm^3 ^[11,12,14]; however, it markedly decreases for TV bigger than 1.5 cm^3 ^although DEC treatment is repeated several times [13]. In clinical studies, DEC effectiveness decreases when TV ≥ 8 cm^3 ^[9,10,15]. It is interesting that 1.5 cm^3 ^is near to V_i_, fact that may suggest that DEC treatment is effective for TV below V_i_, indicating that is important to know this TV in TGK. V_i _may be a criterion of application for this therapy. We suggest to apply electrotherapy for TV below V_i_.

### DEC-treated fibrosarcoma Sa-37 TGK for TG2

In TG2, REG-IIa (from V_o _up to V_min_) is related to the rapid tumor inhibition resulting from DEC cytotoxic action, and REG-IIb (from V_min _up to V_f_) represents the tumor prevalence (tumor re-growth). However, FDTV-TV plot reveals that FDTV first decreases up to FDTV_min _and then increases with decreasing TV in REG-IIa. This may suggest that in this region the tumor self-organizes whereas its volume decreases, indicating that DEC dose is not effective, an aspect not addressed in the literature. As a result, FDTV tends to 0.000068 cm^3^/days corresponding to V_min_, from which TGK triggers.

Tumor destruction (when both TV and FDTV decrease) is caused by DEC cytotoxic action, which induces toxic products in the tumor, generated by electrochemical reactions [19], and it potentiates humoral and cellular components of the immune system [20]. At this time interval, necrosis, apoptosis, chronic inflammation, polymorphous nuclear, monocytes, vascular congestion, and the activation of macrophages and T lymphocytes have been observed [7-15,18-21].

Tumor self-organization is not observed in the TV plot and occurs when FDTV changes of slope independently of the decrease of TV. This timing may occur because the DEC dose used does not induce significant damage to the tumor. As a result, the tumor potentiates its existing mechanisms and/or generates other new mechanisms for its own protection, growth, and metastasis processes in order to reach its maximum survival. This second process can also be explained from the point of view of the complexity theory because the tumor is self-organized and new emergent variables appear [35-38]. This self-organization process of the tumor dominates the process of tumor destruction caused by DEC action, with TV reaching V_min _and consequential tumor re-growth (REG-IIb).

V_min _observed in the TV plot for TG2 is very important from a therapeutic point of view because when TV reaches this value, DEC should be repeated [9,10,13]; however, the results shown in this study indicate that the tumor is self-organized when it reaches V_min_. For us, the existence of FDTV_min _(corresponding to TV = 0.376 cm^3^) on the FDTV-TV plot is surprising because this tumor self-organization process is not observed in the TV plot and therefore its explanation is not possible from this plot. This is relevant, at the therapeutic level because DEC stimulus alone or combined should be repeated when the TV reaches this value.

This procedure may be implemented in practice through two possible ways: 1) by weekly measuring (once or twice) the TV during the first three months after DEC treatment by means of a vernier caliper (for superficial tumors) or ultrasound (for visceral tumors) and 2) by knowing the tumor relaxation time (T_rt_) of a small sample treated with DEC by means of Nuclear Magnetic Resonance method.

In the first way, we observe a significant decrease of TV in DEC treated patients during the first three months, after this time, a tumor re-growth is observed if the dose is not effective [15]. We suggest two measurements/week of TV to obtain various experimental points in the first three months of observation so that the values of the parameters: α, β, γ, and *i*_*o *_can be calculated knowing the values of V_o _and TV on the first four measurements. Then, a numerical method is used to solve a non-homogeneous system of four non-linear equations with these four unknown parameters. This is possible because MGE has a good prediction capability to describe both unperturbed and perturbed tumor growths [17]. We can predict the temporal behavior of TV (TV plot) and of its derived (FDTV plot) once the values of these four parameters are well-known and then estimate FDTV_min _in a FDTV-TV plot. If FDTV changes the sign of its slope (positive to negative) although TV continues increasing, we suggest to repeat this therapy and/or to combine it with another therapeutic procedure, as shown in this study. Therefore, we do not recommend the use Tomography Axial Computerized and Imaging Nuclear Magnetic Resonance, because of their high costs and the regulatory norms established for the use of each one of these imaging techniques.

In the second way, the knowledge of T_rt _is important because we know the time for which the tumor recovers after DEC treatment, and the times that DEC treatment should be repeated in order to the tumor is not self-organize (for example, at a time smaller than T_rt_). The knowledge concerning to these two facts will allow us to determine the exact time at which the DEC should be repeated, and as a result, it will allowed one to avoid unnecessary DEC stimulus to the patient. The tumor self-organization process is slower if the duration of the DEC cytotoxic effect induced into the tumor is greater than T_rt_. In a previous study, we corroborate theoretically that DEC effectiveness increases with the increase of the duration of DEC cytotoxic effect induced into the tumor [17]. The introduction of any of these two possible ways in our experiments will lead to a high antitumor effectiveness, which suggests that our future researchers should take this fact into account.

### DEC-treated fibrosarcoma Sa-37 TGK for TG3

In TG3, REG-IIc (when TV and FDTV both rapidly decrease) may be explained from a biological point of view by DEC cytotoxic action, as we propose above. It should be noted that in the FDTV-TV plot, just before the tumor reaches V_d_, there is a change of slope for FDTV with V_id_, implicating that other antitumor mechanisms have been activated (e.g., the activation of cellular and humoral components of the immune system mentioned above and others unreported until now). In contrast to TG2, in TG3, this change of slope for FDTV does not change its negative sign between V_o _and V_d_.

The net rate of the antitumor processes involved between V_o _and V_id _is higher than that resulting from other antitumor processes induced between V_id _and V_d_. From a biophysical point of view, this indicates the existence of at least two other unknown main antitumor mechanisms, which can occur simultaneously. Each one of these mechanisms has its own time constant, in agreement with previous reports [11]. As a result of these antitumor mechanisms, the tumor is completely destroyed (or reversible). This is corroborated, as TV and FDTV tend to zero when TV is smaller than V_d_; in agreement with our results [17].

The fact that the complete TGK for TG3 is a closed loop suggests the reversibility of the tumor. We believe that this is true if TV is comprehended between V_m _and V_i_. This fact corroborates the above discussion regarding the goal of V_id _in DEC treatment. Some additional experiments are required to prove this statement.

This loop shows that REG-I of TGK (before DEC treatment) and REG-II (after DEC treatment) are asymmetric for all Δt values. The linear fits of these two regions suggest that the slope of the curve for REG-II is 1.37 times higher than that for REG-I, a fact that corroborates that the TV regression rate is proportional to the rate of growth, in agreement with the Norton-Simon hypothesis [39]. Prior to this study, we think that these rates are equal.

This new paradigm forces us to reconsider our knowledge and to modify our traditional approach to research and treatment. This statement is relevant for ET because it completely changes the conception of cancer treatment. The actual idea behind *in vitro *and *in vivo *studies is to treat the tumor and then to observe its evolution, which is not known to priori [7-15,18-21]. However, the existence of V_d _establishes that fibrosarcoma Sa-37 tumors have a DEC threshold for which the tumor is completely destroyed, as demonstrated experimentally and theoretically for Ehrlich and fibrosarcoma Sa-37 tumors [14,17], in agreement with other studies [9,10,18,21,33]. This is possible if we establish an explicit dependence of V_d _as a function of the parameters of Equation 7, the host type, and the ET parameters (dosage and exposure time of DEC, electrode array, and times that DEC is repeated). This is very complex at the experimental and theoretical levels; however, mathematical modeling may be a useful tool for finding an approximate solution (analytical or numerical) to this problem. Such modeling will lead to further improvement in the treatment of solid tumors, and it can also help guide treatment decisions for therapists treating patients (or animals) with this disease. In addition, this statement will contribute to standardizing this therapy.

### New predictions and hypothesis for TGK

In the physical sciences, mathematical theory and experimental investigation have always worked together. Mathematical theory can help to direct experimental research, while the results of experiments help to refine the modeling [2]. This is precisely one of the intentions of this manuscript.

**Figure 9:**
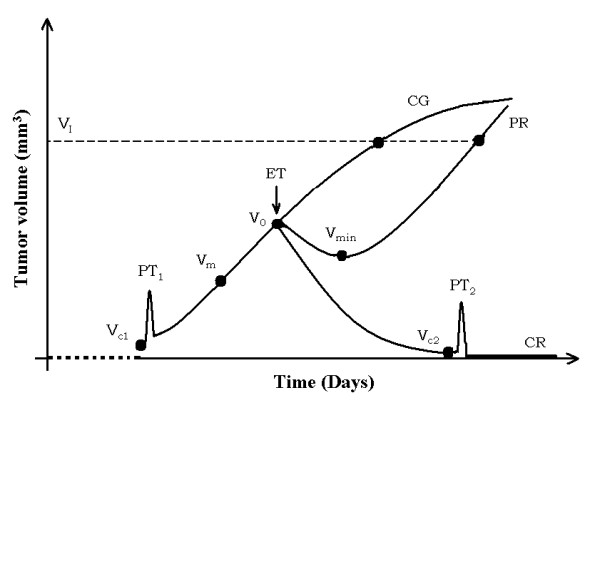
**Schematic representation of TGK**.

**Figure 10:**
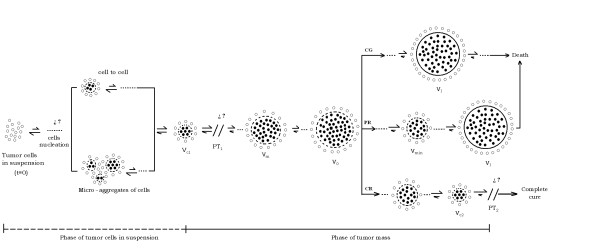
**Representation equivalent of TGK from a biophysical point of view**.

Although Equation 5 (or 7) does not reveal other information, we can propose hypothesis-testing (or hypothesis-generating) methods from it and our experimental observations. The fact that in REG-I of TGK, specifically in SI, for all experimental groups, Ehrlich and fibrosarcoma Sa-37 solid tumors are not observed below V_ob _(tumor cells in suspension) and are observed above V_ob _(solid tumor or tumor mass) may suggest the existence of a phase transition. It is more evident for these tumor types in REG-II of TGK for TG3 when the solid tumor passes from its active phase (below V_d_) to the phase in which the tumor is completely destroyed due to DEC action (above V_d_) [14]. In both cases, these transitions are named PT_1 _and PT_2_, respectively, as schematically represented in Figure 9 and Figure 10. This is also supported if we remember that a phase transition has the characteristic of taking a medium with given properties and transforming some (or all) of it into a new medium with new properties (i.e., the transformation of a thermodynamic system from one phase to another) [40,41].

We know from thermodynamics that at the phase transition point, physical properties may undergo abrupt changes: for instance, the volumes of the two phases may be vastly different, as observed in SI (below and after V_ob_) and REG-II in TG3 (below after V_d_), a fact that could suggest the existence of a critical TV in SI, V_c1_, and another in REG-II in TG3, V_c2_, as schematically represented in Figure 9 and Figure 10. We believe that when V_c1 _(V_c2_) is reached; the tumor begins to grow (completely destroyed).

It is possible that such a phase transition involves a large amount of energy (a dissipative system) accompanied by fluctuations, chaos, and/or self-organization processes with the presence of emergent variables, in agreement with other authors [5,35-38,40-46].

Several authors have reported various phenomena that occur in SI of TGK, such as: a transition from the tumor avascular phase to the vascular phase (angiogenesis), which is accompanied by fluctuations [5,25-28,31,32]; the existence of a stochastic transition at the change between these two tumor phases [29]; the disruption of normal blood vessels of the organs in which the tumor is developing caused by chaotic growth [25]; the existence of a threshold under which sprouts cannot reach the tumor during the growth of the vascular network [46]; among others. It is interesting that our model reveals that SI is highly non linear, a fact that could be associated with the presence of chaos [5,42,43], in agreement with other authors [5,42,43]. This corresponds with established non-equilibrium thermodynamics, in which systems driven out of equilibrium (as solid tumors and biological systems generally are) often exhibit fluctuations or phase transitions [35,44]. In addition, these systems can develop from disorder (systems known as dissipative) because they are formed and maintained by dissipative processes that take place due to an exchange of energy and matter between the system and its environment, and they disappear if that exchange ceases. From Equation 1 (5 or 6), it may be corroborated that a tumor is a dissipative system because *i*_*o *_is much lower than *i *[17]. The biological processes that are constantly receiving, transforming, and dissipating chemical energy can, and do, exhibit properties of self-organization far from thermodynamic equilibrium [35-38,44,45].

MGE offers information of the global dynamics of unperturbed and DEC treated tumors and therefore only gives a limited understanding about the self-organization processes in TGK. However, we believe that these processes are involved in unperturbed and DEC treated TGK for discussed above and the following facts, which are implicitly in MGE: 1) Self-organization makes sense only in relation to the whole: it is the whole that self-organizes into a multitude of interacting levels. At the same time, the whole cannot sustain its integrity, if the process of self-organization does not work. This suggests that self-organization has an important role in the formation, maintenance, and function of cells, tissues, organs and the complete human body. 2) A key requirement for a self-organizing system is nonlinearity and therefore the self-organizing systems are governed by nonlinear dynamics [47], in agreement with our results. 3) Gompertzian dynamics emerges as a result of the fractal-stochastic dualism, which is a universal natural law of biological complexity [48], in agreement with Brú *et al*. [49]. 3) System changes from non-order to order, from low-grade order to advanced order, basis on the principle of auto-organization adaption [50]. 4) Cancer is a reflection of a failing system; preventive steps should involve rebalancing the entire system through lowering of disorderly complexity, entropy, and optimizing self-organization with orderly complexity [51]. 5) The malignant tumor is a complex system and therefore this complexity expresses its functionality and reflects a high degree of resilience and robustness to environmental challenges through their self-adaptation and internal self-organization [51]. 6) The process of tumor cell growth, invasion and metastasis involves a self-organized cascade of multiple tumor-host and tumor-immune interactions [52]. Self-organization might be a general principle in cellular organization and an elegant, efficient way to optimally organize cellular structures [53]. 7) Self-organization occurs when a real system evolves toward a higher differentiation from its initial state (or pre-*system *phase) [51]. These two phases are revealed with MGE: pre-tumor phase (below V_ob_) and solid tumor phase (above V_ob_). Also, this differentiation is observed in our pathological studies [11-15], and it is the cause of the aggressiveness and difference in the cellular/molecular patterns of the different types of malignant tumors [1]. In spite of these facts and others, more studies at cellular/molecular/atomic/quantum levels and new physic-mathematical approaches are needed to have more meaningful results about the self-organization process in TGK.

During such a phase transition, a tumor either absorbs or releases a fixed (and typically large) amount of energy, which is characteristic of a first-order phase transition. Because energy cannot be instantaneously transferred between the tumor and it's surrounding healthy tissue, first-order transitions are associated with "mixed-phase regimes" in which some parts of the system have completed the transition and others have not. Based on statistical physics, mixed-phase systems are difficult to study, because their dynamics are violent and challenging to control [40].

The hypotheses proposed in this study can doubtlessly be seriously attacked by many; however, this study sets the basis to derive some practical understanding from our diverse (and often, at this time, empirical) experimental and clinical observations in cancer electrotherapy. The availability of powerful computers has already helped to bridge the gap between observations and predictions in many complex problems, and a few attempts have already been made to attack the problem of tumor growth with mathematical models.

We are recognizing biophysics principles that may be broadly applied in developing more useful programs of DEC treatment of solid tumors. To begin to understand the complexity of the proposed system, novel simulations must be developed, incorporating concepts from many scientific areas such as cancer research, statistical mechanics, applied mathematics, and nonlinear dynamical systems.

Our results suggest that the MGE should be modified, or a new mathematical approach should be proposed in order to describe TGK and explain the presence of at least one of these phenomena. These results are, in agreement with Bellomo *et al*. [2], who proposed that "future research will definitely refine and improve the existing models, while the analysis of the inherent mathematical problems will hopefully lead to new mathematics, allowing us to tackle problems presently beyond our technical abilities".

## Conclusion

In conclusion, the modified Gompertz equation is likely to lead to insights within cancer research. Such insights hold promise for increasing our understanding of tumors as self-organizing systems and, the possible existence of phase transitions in tumor growth kinetics, which, in turn, may have a significant impact both on cancer research and on clinical practice.

## List of abbreviations used

ET: electrotherapy; DEC: direct electric current; CG: control group; TG1: treated group 1; TG2: treated group 2; TG3: treated group 3; MGE: modified Gompertz equation; TV: tumor volume; TGK: tumor growth kinetics; REG-I: part of TGK before DEC treatment; REG-II: part of TGK after DEC treatment; REG-IIa and REG-IIb: sub-regions of REG-II for TG2; REG-IIc and REG-IId: sub-regions of REG-II for TG3; FDTV: first derivative of tumor volume; FDTV_max_: maximum FDTV observed in TGK for the CG; FDTV_min_: minimum FDTV observed in TG2; TV plot: tumor volume versus t plot; FDTV plot: time dependence of first derivative of tumor volume plot; FDTV-TV plot: first derivative of tumor volume versus tumor volume; CTV plot: time consecutive dependence of tumor volume plot; V_o_: initial volume at which DEC is supplied; V_ob_: first non-zero value of TV; τ: time delay; T_rt_: tumor relaxation time; V_min_: minimum TV; V_d_: TV from which begins tumor complete destruction; V_id_: inflection point in REG-IIc; V_m_: smallest measurable TV; V_f_: final volume of TGK; t_ob_: time at which V_ob _is observed; V_i_: point of inflection in TGK; V_s_: TV that separates SI and SII; V_ic_: TV that separates SII and SIII; Δt: time step; SI, SII, and SIII are the first, second, and third stages in TGK of the control group, respectively; PT_1_: phase transition between the phases of tumor cells in suspension and a solid tumor; V_c1_: critical volume for which PT_1 _occurs; PT_2_: phase transition between an active solid tumor and a completely destroyed tumor; V_c2_: critical volume for which PT_2 _occurs.

## Competing interests

The authors declare that they have no competing interests.

## Authors' contributions

LEBC planned the study, and participated in its design and coordination, and also assisted with the manuscript. JJGN participated in its design and discussion, and also contributed to the manuscript. ARA and JAGJ organized this study and participated in the elaboration of the software to obtain the results shown. HMCC, MMG, MFS, MVJ, TRG, MAOM, SCAB, FSP, LZO, MCCQ, SES, VCC, IBC, and GSG participated in the design of this study, and contributed to the analysis, interpretation, and discussion of the results. All authors read and approved the final manuscript.

It is important to point out that studies in cancer necessarily require the knowledge of a multidisciplinary group of researchers because cancer is not well-understand, and currently, no therapy that completely cures this illness has been reported.

## Pre-publication history

The pre-publication history for this paper can be accessed here:

http://www.biomedcentral.com/1471-2407/10/589/prepub
